# MRI Radiomic Features: A Potential Biomarker for Progression-Free Survival Prediction of Patients With Locally Advanced Cervical Cancer Undergoing Surgery

**DOI:** 10.3389/fonc.2021.749114

**Published:** 2021-12-14

**Authors:** Mengting Cai, Fei Yao, Jie Ding, Ruru Zheng, Xiaowan Huang, Yunjun Yang, Feng Lin, Zhangyong Hu

**Affiliations:** ^1^ Department of Radiology, The First Affiliated Hospital of Wenzhou Medical University, Wenzhou, China; ^2^ Department of Gynecology, The First Affiliated Hospital of Wenzhou Medical University, Wenzhou, China

**Keywords:** locally advanced cervical cancer, magnetic resonance imaging, radiomics, progression-free survival, MRI

## Abstract

**Objectives:**

To investigate the prognostic role of radiomic features based on pretreatment MRI in predicting progression-free survival (PFS) of locally advanced cervical cancer (LACC).

**Methods:**

All 181 women with histologically confirmed LACC were randomly divided into the training cohort (n = 126) and the validation cohort (n = 55). For each patient, we extracted radiomic features from whole tumors on sagittal T2WI and axial DWI. The least absolute shrinkage and selection operator (LASSO) algorithm combined with the Cox survival analysis was applied to select features and construct a radiomic score (Rad-score) model. The cutoff value of the Rad-score was used to divide the patients into high- and low-risk groups by the X-tile. Kaplan–Meier analysis and log-rank test were used to assess the prognostic value of the Rad-score. In addition, we totally developed three models, the clinical model, the Rad-score, and the combined nomogram.

**Results:**

The Rad-score demonstrated good performance in stratifying patients into high- and low-risk groups of progression in the training (HR = 3.279, 95% CI: 2.865–3.693, *p* < 0.0001) and validation cohorts (HR = 2.247, 95% CI: 1.735–2.759, *p* < 0.0001). Otherwise, the combined nomogram, integrating the Rad-score and patient’s age, hemoglobin, white blood cell, and lymph vascular space invasion, demonstrated prominent discrimination, yielding an AUC of 0.879 (95% CI, 0.811–0.947) in the training cohort and 0.820 (95% CI, 0.668–0.971) in the validation cohort. The Delong test verified that the combined nomogram showed better performance in estimating PFS than the clinical model and Rad-score in the training cohort (*p* = 0.038, *p* = 0.043).

**Conclusion:**

The radiomics nomogram performed well in individualized PFS estimation for the patients with LACC, which might guide individual treatment decisions.

## Introduction

Cervical cancer is one of the most common cancer in women worldwide and an important cause of cancer-related death among women (1). In developing countries, screening has not yet been fully universal, and the incidence and mortality of cervical cancer are still on the rise. In China, most cervical cancer patients are at advanced stages when diagnosed (2). Radical hysterectomy or concurrent chemoradiotherapy is the standard treatment protocol for locally advanced cervical cancer (LACC) (3). Nevertheless, recurrence or metastasis frequently occurred in these patients, with only 50%~60% 5-year survival rate. Thence, pretreatment prediction for the high-risk recurrence or distant metastasis is important for the development of individualized treatment protocols.

Several clinical factors have already been identified as risk factors in cervical cancer patients, including International Federation of Gynecology and Obstetrics (FIGO) stage, lymph node metastasis (LNM), lymph vascular space invasion (LVSI), and depth of invasion (4, 5). Nevertheless, even if the clinical stage and treatment plan of the patient are similar, the clinical outcome can vary widely. These findings imply that the present prognostic model could not provide adequate prognostic information and correctly assess the intrinsic heterogeneity of tumors. Hence, new prognostic biomarkers are required for individual treatment.

Pretreatment magnetic resonance imaging (MRI) can supply more details about tumor heterogeneity than tissue samples and assist in determining the tumor size, location, degree of invasion into adjacent organs, and LNM (6, 7). The emerging radiomics holds great potential for facilitating better clinical decision-making. Radiomics refers to the conversion of medical images into mineable high-dimensional data *via* automatic high-throughput extraction of data characterization algorithms (8, 9). The main function of radiomics is that the image data-mining method can detect the intrinsic heterogeneity of tumors, unidentifiable by radiologists, and provide decision support noninvasively for oncology at low cost (10, 11). According to previous studies, radiomics features could predict the survival outcomes and recurrence, evaluate tumor subtype and stage, monitor therapeutic response, and detect LNM or distant metastasis (12–14). It is unknown whether radiomics signatures of pretreatment MRI can predict progression-free survival (PFS) in LACC patients who received radical hysterectomy without preoperative neoadjuvant chemotherapy.

Therefore, the purpose of our study is to develop and validate a noninvasive radiomics signature based on pretreatment MRI for the PFS prediction in patients with LACC.

## Materials and Methods

### Patients

We identified 181 consecutive women with LACC who underwent surgeries following pretreatment MRI using a 3.0-T scanner at our institution between January 2011 and February 2017 (to ensure a minimum follow-up of 3 years). The inclusion criteria were as follows (**Supplementary Figure S1**): (i) patients who underwent radical hysterectomies and pelvic lymphadenectomies; (ii) patients who have not received treatment before surgery and the clinicopathological data are complete; and (iii) sagittal T2-weighted imaging (T2WI) and axial diffusion-weighted imaging (DWI) were performed less than the 2-week period before surgery. The exclusion criteria were as follows: (i) lesions invisible on axial DWI or sagittal T2WI; (ii) poor image quality due to the movement of the patient during examination or the chemical shift artifact of the gas in the colorectum; (iii) patients who underwent preoperative therapies; and (iv) patients with other cancers at the same time.

### Follow-Up and Prognosis Evaluation

Information was collected from telephone consultations, outpatient medical records, and social security death indices. In our study, the endpoint event was PFS, which was defined as the time from the date of surgery until any recurrence (local or distant recurrence, metastasis). Clinical follow-up was every 3 months for the first 2 years, every 6 months for the next 3 years, and annually thereafter. Gynecological examinations, cervical cytology, and imaging tools such as computed tomography (CT), MR, and positron emission tomography (PET)/CT imaging were used to evaluate the patients during the follow-up.

### MRI Scan Acquisition and Tumor Segmentation

All patients underwent pelvic 3.0-T MRI scans (Signa EXCITE; GE Medical Systems, 3200N, Grandview Blvd, Waukesha, WI 53188, USA; or Achieva, Philips Healthcare, The Netherlands), using a 16-channel phased-array encoding abdominal coil. The scanning range was set to cover the entire pelvis from the level of the anterior superior iliac spine to the inferior level of the symphysis pubis. Patients had to fast at least 6 h before the examination. The standard pelvic MR scan protocol was used in this retrospective study, including multiple b-value DWI (repetition time (TR)/echo time (TE) = 3,625/74 ms, field of view (FOV): 420 × 420 mm, matrix: 256 × 128, slice thickness/gap = 5 mm/6 mm, b-values: 0.700 s/mm^2^), sagittal T2-weighted fat suppression (FS) images: (TR/TE = 3,200/106 ms, FOV = 320 × 320 mm, matrix = 320 × 224, slice thickness/gap = 4 mm/5 mm); axial T2-weighted fat suppression (FS) images: (TR/TE = 3,600/104 ms, FOV = 380 × 380 mm, matrix = 320 × 224. slice thickness/gap = 5 mm/6 mm); coronal T2-weighted fat suppression (FS) images: (TR/TE = 3,400/107 ms, FOV = 360 × 360 mm, matrix = 320 × 192, slice thickness/gap = 4 mm/5 mm); and axial T1-weighted imaging (T1WI): (TR/TE = 245 ms/2 ms, FOV: 380 × 380 mm, matrix: 384 × 180, slice thickness/gap = 5 mm/6 mm).

**Figure 1:**
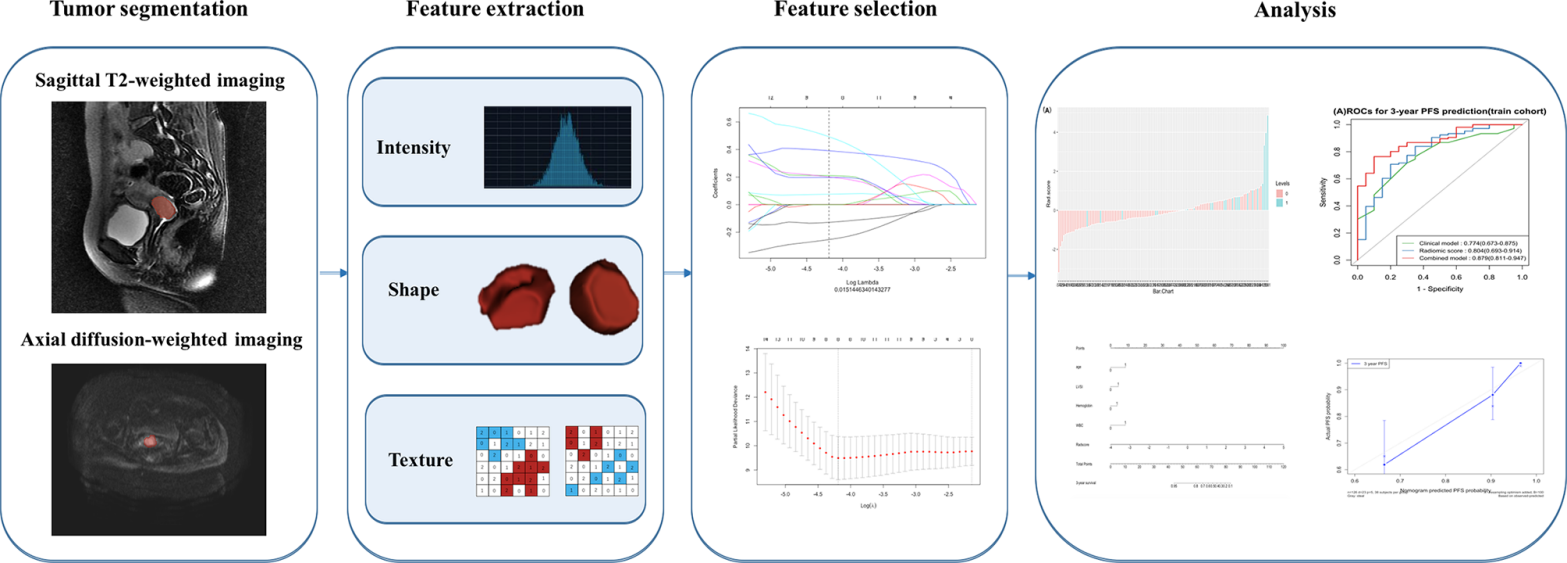
The radiomics workflow.

Pelvic MRI Digital Imaging and Communications in Medicine (DICOM) original images of all patients were downloaded from the Picture Archiving and Communication System (PACS) and uploaded into the ITK-SNAP (open source software; www.itksnap.org) for three-dimensional manual segmentation of MR images (15). The regions of interest (ROIs) of the entire tumor were manually outlined layer by layer by a radiologist with 5 years of experience in gynecological imaging, and the results were verified by a senior radiologist with 15 years of work experience. The radiomics workflow is displayed in **Figure 1**.

### Feature Extraction

Before feature extraction, the image was preprocessed, including resampling the MR image to a 1 × 1 × 1-mm^3^ voxel size and normalizing the image gray scale to 0 to 255. The purpose of image preprocessing is to reduce heterogeneity bias caused by different equipments and scanning parameters. Radiomic feature extraction was implemented in Artificial Intelligence Kit Version 3.0.0.R, which is a commercial software of GE Healthcare. We extracted 396 radiomic features of tumor on T2WI and DWI, respectively, and a total of 792 quantitative features for each patient. The features were divided into four groups: (I) intensity histogram (n = 84), (II) morphology (n = 40) and (III) texture (n = 668), including (a) gray-level co-occurrence matrix (GLCM, n = 288), (b) gray-level run length matrix (GLRLM, n = 344), and (c) Haralick (n = 36).

### Feature Selection

High-dimensional extraction produced various radiomic features, but not all of them were significantly associated with PFS in LACC. To develop the radiomics model, we designed a two-step procedure for dimensionality reduction and selection of radiomic features. We applied the least absolute shrinkage and selection operator (LASSO) algorithm jointly with the Cox survival analysis to select the importantly prognostic features in the training cohort (16, 17). Then, the multiple-feature-based radiomic signatures were created for predicting patients’ PFS in the training cohort. The LASSO Cox regression model analysis was completed by the “glmnet” package (18, 19).

### Building and Validation of the Radiomics Signature

The radiomics score (Rad-score, which was defined as the radiomics signature in the current study) was computed in the training cohort by the LASSO Cox regression model analysis. Above all, we evaluated the potential relationship of the Rad-score with PFS in the training cohort and then tested it in the validation cohort. In addition, all patients were divided into high-risk and low-risk groups according to the optimum cutoff value of the Rad-score by applying X-tile software (20). The relationship of the radiomic risk score with PFS was assessed in the training cohort and then verified in the validation cohort by using Kaplan–Meier analysis and log-rank test.

### Construction of an Individualized PFS Prediction Model

The univariate Cox analysis was used to determine clinicopathologic factors associated with PFS in all patients (n = 181). LASSO Cox regression model analysis was applied to select variables with a *p* value < 0.1 in the univariate Cox analysis. Except the Rad-score, a clinical model which incorporated only the independent clinicopathologic risk factors was also built to predict the 3-year PFS. The prognostic performance of the models was measured by Harrell’s concordance index (C-index), C-index = 0.5 describes a random prediction, and C-index = 1.0 implies a perfect prediction ability (21). Since the C-index method may be wrong when predicting a fixed time point (22), we also use the time-dependent area under the receiver operating characteristic (ROC) analysis to assess the t-year risk of an event (23, 24).

To provide the clinician with a quantitative approach to predict patients’ probability of 3-year PFS, and to show the incremental value of the Rad-score to the clinicopathologic risk factors, we further built a combined nomogram (combined model) as an individualized PFS prediction model that incorporated both the Rad-score and clinicopathologic risk factors for PFS prediction. The prognostic performance of the nomogram was estimated by C-index and ROC analysis. Besides, calibration curves were used to compare the predicted PFS with the actual PFS.

### Statistical Analysis

Statistical analysis was executed by R software (version 3.6.3) and SPSS (version 23.0). Rad-scores were divided into two groups according to the cutoff value selected by X-tile. In addition, the continuous clinical variables were converted into categorical variables on the basis of cutoff values, which were determined by ROC analysis or routine cutoff points (for size). Differences in distributions between the training and validation cohorts were assessed with the chi-squared test as appropriate. A quantitative comparison of the area under the curve (AUC) was made with the Delong test (25). The glmnet package was adopted for running LASSO–Cox. The survival package was adopted for building the Cox proportional risk model, drawing the Kaplan–Meier analysis, and calculating the C-index. The rms package was used for nomograms and calibration curves. All two-sided *p* values less than 0.05 were considered significant.

## Results

### Patient Characteristics

The clinicopathologic factors of all patients are summarized in **Table 1**. Of the 181 patients included in the study, the mean age of the patients was 50.67 ± 10.81 years. Disease recurred in 33 of 181 patients (18.2%). The median follow-up time was 50.5 months (40.75–66.0) for the training cohort and 48.0 months (40.0–66.0) for the validation cohort. There were no significant differences between the training cohort and the validation cohort in clinicopathologic features (*p* = 0.146–0.714).

**Table 1:** Patient characteristics in the training and validation cohorts.

Characteristics		Training cohort (n = 126)	Validation cohort (n = 55)	p value
Age (years)				0.714
≥54.50		65 (51.59%)	30 (54.55%)	
<54.50		61 (48.41%)	25 (45.45%)	
FIGO stage				0.357
IB1		43 (34.13%)	24 (43.64.09%)	
IB2		10 (7.93%)	6 (10.91%)	
IIA1		54 (42.86%)	16 (29.09%)	
IIA2		19 (15.08%)	9 (16.36%)	
Size(cm)				0.539
2~4		97 (76.98%)	40 (72.73%)	
>4		29 (23.02%)	15 (27.27%)	
LVSI				0.360
Positive		35 (27.78%)	19 (34.55%)	
Negative		91 (72.22%)	36 (65.45%)	
PLN				0.409
Positive		21 (16.67%)	12 (21.82%)	
Negative		105 (83.33%)	43 (78.18%)	
Differentiation				0.758
Low grade		5 (3.97%)	1 (1.82%)	
Middle grade		51 (40.48%)	23 (41.82%)	
High grade		70 (55.56%)	31 (56.36%)	
Pathological type				0.260
SCC		110 (87.30%)	52 (94.54%)	
Adenocarcinoma		10 (7.94%)	1 (1.82%)	
Other		6 (4.76%)	2 (3.64%)	
SCCA(μg/L)				0.887
≥2.05		70 (58.33%))	28 (57.14%)	
<2.05		50 (41.67%	21 (42.86%)	
Albumin (g/L)				0.215
≥41.25		104 (82.54%)	41 (74.55%)	
<41.25		22 (17.46%)	14 (25.45%)	
Hemoglobin (g/L)				0.409
≥145.5		11 (8.73%)	7 (12.73%)	
<145.5		115 (91.27%)	48 (87.27%)	
Platelet (10^9^/L)				0.653
≥296		29 (23.02%)	11 (20%)	
<296		97 (76.98%)	44 (80%)	
WBC (10^9^/L)				0.236
≥7.465		39 (30.95%)	22 (40%)	
<7.465		87 (69.05%)	33 (60%)	

FIGO, International Federation of Gynecology and Obstetrics; LVSI, lymph vascular space invasion; LNM, lymph node metastasis; SCC, squamous cell carcinoma; SCCA, squamous cell carcinoma antigen; WBC, white blood cell.

### Feature Selection, Radiomics Signature Building and Validation

The LASSO Cox regression model was used to build a prognostic Rad-score. Eight potential predictors, six features from T2WI, and two features from DWI were included in the training cohort. Finally, the Rad-score was constructed based on the eight features, and the calculation formula is as follows: Rad-score = -0.253 × T2WI_MinIntensity + 0.210 × T2WI_ClusterShade + 0.076 × T2WI_GLCM-IDM + 0.201 × T2WI_RLM-LongRunEmphasis × T2WI_RLM-LongRunHighGreyLevelEmphasis + 0.391 × T2WI_LowIntensityLargeAreaEmphasis + 0.198 × DWI_HighIntensitySmallAreaEmphasis + 0.493 × DWI_SmallAreaEmphasis. Distributions of the Rad-score in the training and validation cohorts are displayed in **Supplementary Figure S2**.

**Figure 2:**
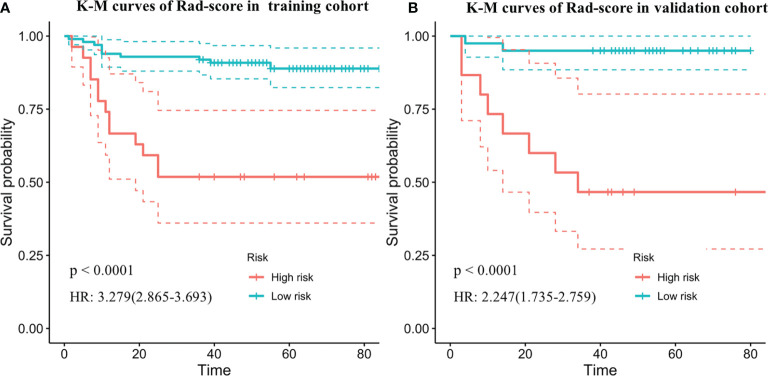
Kaplan–Meier survival curves of progression-free survival according to the Rad-score in the training cohorts **(A)** and independent validation data set **(B)**. Shadows represent 95% confidence interval. HR, hazard ratio.

**Figure 3:**
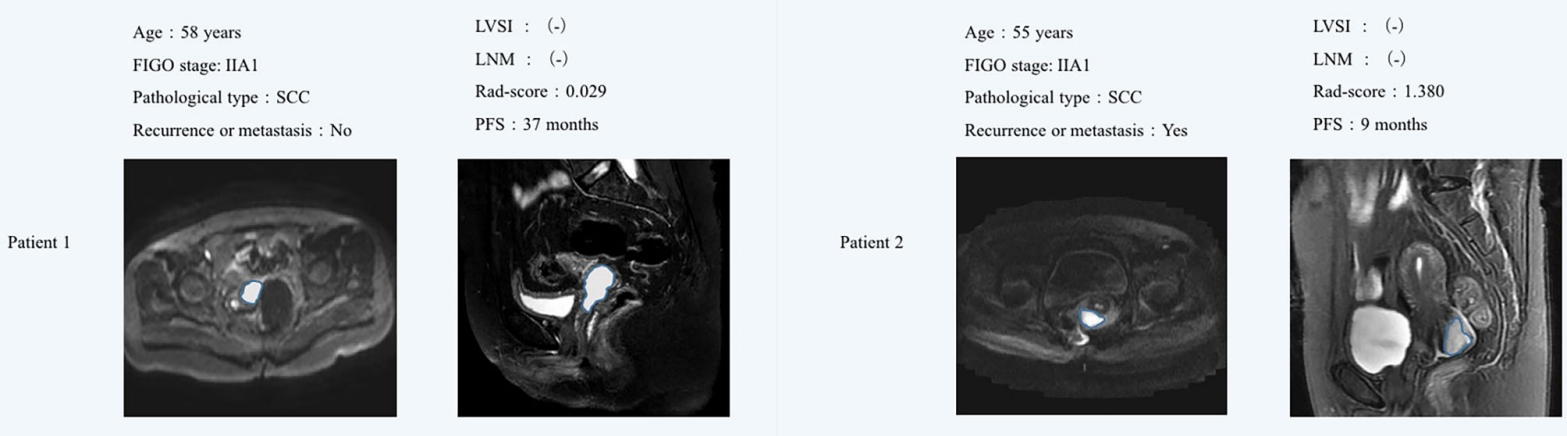
MR images showed that the sagittal T2WI and axial DWI lesions were cervical cancer. Although patient 1 and patient 2 had similar clinicopathological characteristics, their Rad-score were significantly different. Patient 1 did not find obvious signs of recurrence or metastasis after 37 months of postoperative follow-up, while the Patient 2 relapsed 9 months after surgery. The Rad-score of patient 2 was significantly higher than that of patient 1. FIGO, International Federation of Gynecology and Obstetrics; LVSI, lymph vascular space invasion; LNM, lymph node metastasis; SCC, squamous cell carcinoma; PFS, progression-free survival.

The optimum cutoff generated by X-tile was 0.543. According to the optimal cutoff value, patients were classified into high-risk (Rad-score ≥ 0.543) and low-risk (Rad-score < 0.543) groups. A Kaplan–Meier analysis (**Figure 2**) was demonstrated that the patients in the high-risk group had shorter PFS than did the low-risk group in the training [hazard ratio (HR) = 3.279, 95% CI: 2.865–3.693, *p* < 0.0001] and validation cohorts (HR = 2.247, 95% CI: 1.735–2.759, *p* < 0.0001). **Figure 3** shows typical patients who had similar clinicopathological characteristics, but their PFS time was significantly different (9 vs. 37 months). The Rad-score of patient 2 was significantly higher than that of patient 1 (1.380 vs. 0.029). In the LASSO Cox regression analysis, the Rad-score yielded a C-index of 0.778 [95% confidence interval (CI): 0.699–0.858] for the training cohort (**Table 2**). The favorable prognostic performance of the Rad-score was further confirmed in the validation cohort (C-index, 0.816; 95% CI: 0.673–0.958).

**Table 2:** Model performance on predicting 3-year PFS.

Models	Cohorts	C-index (95% CI)	AUC (95% CI)	ACC (95% CI)	Sensitivity (95% CI)	Specificity (95% CI)
**Clinical model**	Training	0.778 (0.699–0.858)	0.774 (0.673–0.875)	0.714 (0.711–0.717)	0.717 (0.631–0.803)	0.700 (0.499–0.901)
	Validation	0.816 (0.673–0.958)	0.707 (0.513–0.902)	0.764 (0.757–0.770)	0.800 (0.683–0.917)	0.600 (0.296–0.904)
**Radiomic score**	Training	0.756 (0.650–0.861)	0.804 (0.693–0.914)	0.722 (0.719–0.725)	0.708 (0.621–0.794)	0.800 (0.625–0.975)
	Validation	0.803 (0.690–0.915)	0.795 (0.653–0.937)	0.764 (0.757–0.770)	0.756 (0.630–0.881)	0.800 (0.552–1.048)
**Combined model**	Training	0.821 (0.746–0.896)	0.879 (0.811–0.947)	0.786 (0.783–0.788)	0.764 (0.683–0.845)	0.900 (0.769–1.031)
	Validation	0.829 (0.699–0.959)	0.82 (0.668–0.971)	0.855 (0.850–0.859)	0.867 (0.767–0.966)	0.800 (0.552–1.048)

PFS, progression-free survival; C-index, Harrell’s concordance indices; CI, confidence interval; AUC, area under the curve; ACC, accuracy.

### Univariate Cox Analysis of the Risk Factors for PFS

In addition to Rad-score, we included a total of 12 clinicopathologic factors into the univariate analysis (**Table 3**), and the results showed that age (HR = 0.511, 95% CI: 0.247–1.053, *p* = 0.069), LVSI (HR = 2.286, 95% CI: 1.151–4.538, *p* = 0.018), squamous cell carcinoma antigen (HR = 0.427, 95% CI: 0.182–1.005, *p* = 0.051), albumin (HR = 0.530, 95% CI: 0.252–1.113, *p* = 0.091), hemoglobin (HR = 5.393, 95% CI: 2.560–11.363, *p <* 0.0001), white blood cell (WBC) (HR = 2.233, 95% CI: 1.128–4.420, *p* = 0.021), and Rad-score (HR = 2.647, 95% CI: 2.002–3.501, p < 0.0001), would be identified as candidate risk factors (*p* < 0.1) into the LASSO Cox regression analysis.

**Table 3:** Univariate Cox analysis of risk factors for PFS in all patients.

Characteristics	Univariate analysis		
	Hazard ratio	95% CI	p value
Age			
≥54.5 years versus <54.5 years	0.511	0.247–1.053	0.069
Size			
2~4 cm versus >4 cm	0.851	0.396–1.831	0.680
FIGO stage			
IB1	Reference		
IB2	0.352	0.045–2.724	0.317
IIA1	1.337	0.614–2.912	0.464
IIA2	1.369	0.506–3.701	0.536
LVSI			
positive versus negative	2.286	1.151–4.538	0.018
PLN			
positive versus negative	1.748	0.813–3.762	0.153
Differentiation			
Low grade	Reference		
Middle grade	0.779	0.105–5.791	0.807
High grade	0.653	0.315–1.355	0.253
Pathological type			
SCC	Reference		
Adenocarcinoma	0.473	0.064–3.473	0.462
Other	2.469	0.751–8.112	0.137
SCCA (μg/L)			
≥2.05 versus <2.05	0.427	0.182–1.005	0.051
Albumin (g/L)			
≥41.25 versus <41.25	0.530	0.252–1.113	0.094
Hemoglobin (g/L)			
≥145.5 versus <145.5	5.393	2.560–11.363	<0.0001
Platelet (10^9^/L)			
≥296 versus <296	1.088	0.472–2.508	0.843
WBC (10^9^/L)			
≥7.465 versus <7.465	2.233	1.128–4.420	0.021
Rad-score	2.647	2.002–3.501	<0.0001

PFS, progression-free survival; FIGO, International Federation of Gynecology and Obstetrics; LVSI, lymph vascular space invasion; LNM, lymph node metastasis; SCC, squamous cell carcinoma; SCCA, squamous cell carcinoma antigen; WBC, white blood cell; CI, confidence interval.

### Assessment the Performances of Various Models in 3-Year PFS Prediction

**Figure 4:**
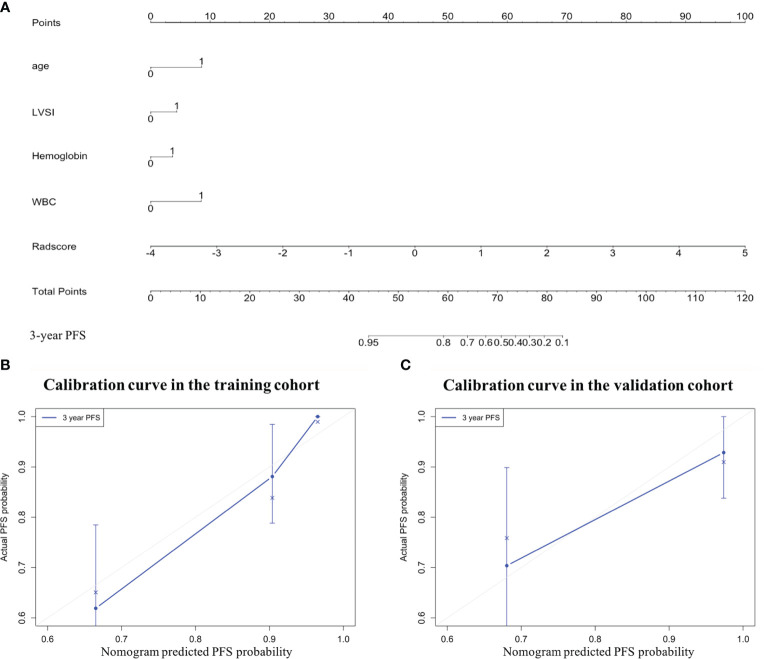
The combined nomogram was developed to predict the risk of 3-year progression-free survival (PFS) of patients with locally advanced cervical cancer undergoing surgery. **(A)** The combined nomogram that integrates the Rad-score with the clinicopathologic features in the training data set. Calibration curves of the combined nomogram in the **(B)** training and **(C)** validation cohorts. The diagonal gray line represents a perfect evaluation, while the blue line represents the actual performance of the nomogram. A closer fit to the diagonal gray line indicates a better assessment. LVSI, lymph vascular space invasion; WBC, white blood cell.

The LASSO Cox regression model analysis showed that age, LVSI, hemoglobin, and WBC were finally selected and integrated into a clinical model (**Figure 4**). The performance of the clinical model for 3-year PFS prediction yielded a C-index value of 0.778 (95% CI: 0.699–0.858) in the training cohort and 0.816 (95% CI: 0.673–0.958) in the validation cohort (**Table 2**). The combined nomogram (**Figure 4**) integrating the radiomic score and the above four clinicopathologic factors demonstrated a better discrimination both in the training (C-index, 0.821; 95% CI: 0.746–0.896) and in the validation cohort (C-index, 0.829; 95% CI: 0.699–0.959) when compared with radiomic score or clinical model alone (**Table 2**).

**Figure 5:**
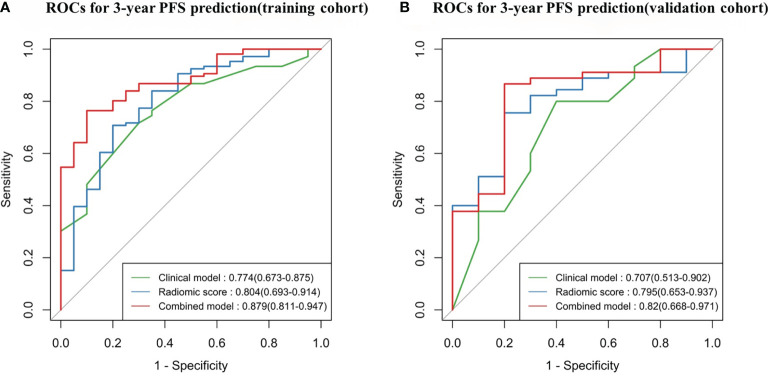
Time-dependent receiver operating characteristic (ROC) curves comparing the predictive capacity of the three models. **(A)** Training cohort, N = 126. **(B)** Validation cohort, N = 55. PFS, progression-free survival.

**Figure 6:**
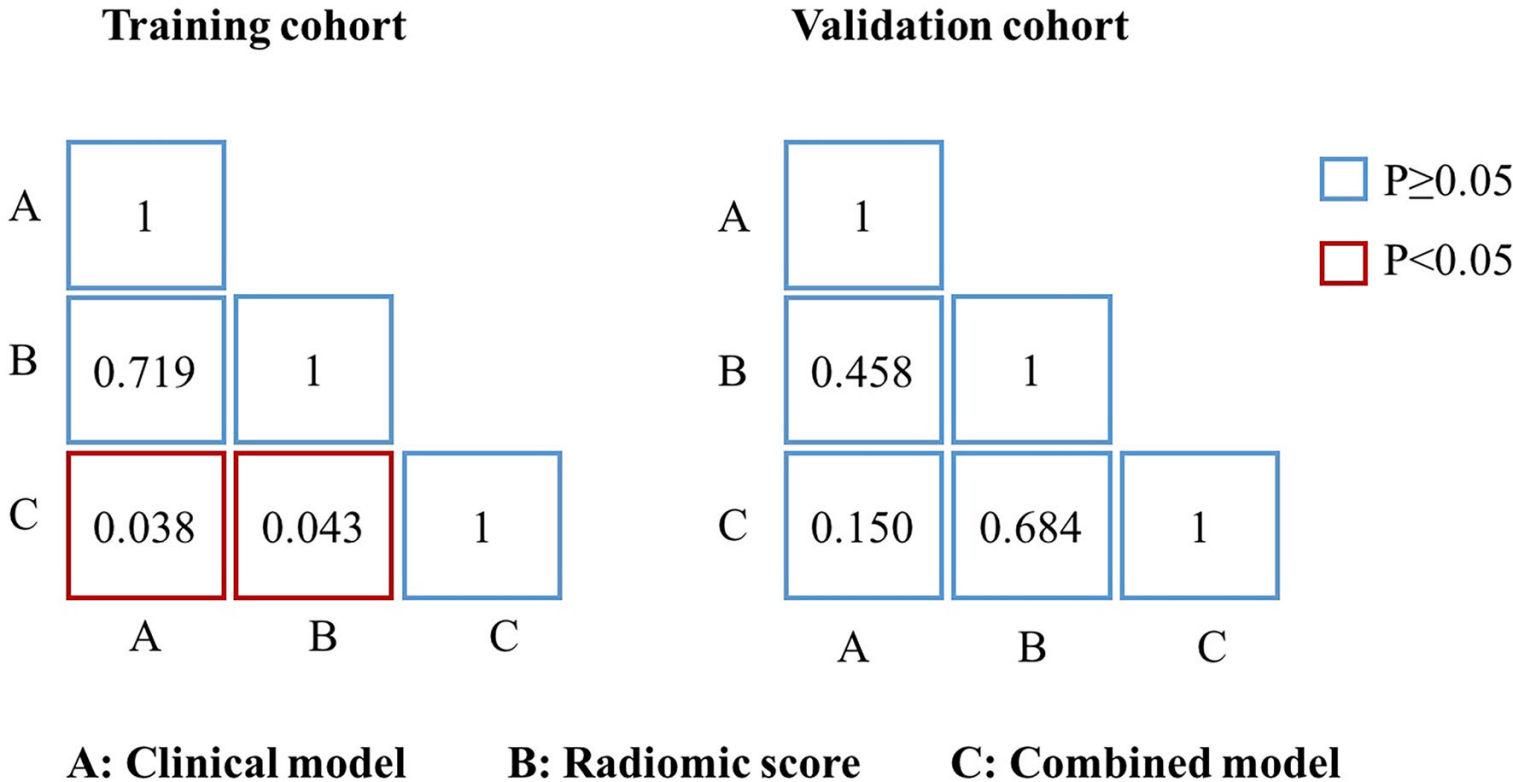
Delong test between different models. p-value of the Delong test between any two models.

In the training cohort (**Table 2** and **Figure 5A**), the radiomics score (AUC, 0.804 [95% CI: 0.693–0.914]) showed a comparable prognostic performance with the clinical model (AUC, 0.774 [95% CI: 0.673–0.875]), with a *p* value of 0.719 (**Figure 6**). The combined nomogram was shown to be with the highest AUC value (0.879, [95% CI: 0.811–0.947]), demonstrating a significant improvement in PFS prediction compared to the clinical model or the radiomics score (*p* = 0.038, *p* = 0.043, respectively) (**Figure 6**). In the validation cohort (**Table 2** and **Figure 5B**), the clinical model yielded an AUC of 0.707 (95% CI: 0.513–0.902). Although the radiomics score (AUC, 0.795 [95% CI: 0.653–0.937]) showed improvement compared with the clinical model, the Delong test found that no significant difference was shown between the AUCs (*p* = 0.458) (**Figure 6**). The combined nomogram (AUC, 0.820 [95% CI: 0.668–0.971]) also showed improvement over the clinical model or the radiomics score for the PFS prediction in the validation cohort. However, the difference was not significant (*p* = 0.150, *p* = 0.684, respectively) (**Figure 6**).

## Discussion

In this study, we developed and validated an MRI-based Rad-score for noninvasive PFS prediction in patients with LACC undergoing surgery. The study demonstrated that the Rad-score was significantly related with 3-year PFS in both the training and validation cohorts. The Rad-score stratified the patients into low-risk and high-risk groups, and the Kaplan–Meier analysis showed that the patients in the high-risk group portended a worse prognosis with shorter PFS than did the low-risk group in the training (HR = 3.279, 95% CI: 2.865–3.693, *p* < 0.0001) and validation cohorts (HR = 2.247, 95% CI: 1.735–2.759, *p* < 0.0001). The performance of the combined nomogram which integrated Rad-score and significant clinicopathologic parameters was demonstrated to have better performance than the Rad-score or clinical model alone in the PFS prediction. Furthermore, the nomogram showed a satisfactory discrimination performance, with C-indexes of 0.821 and 0.829 in the training and validation cohorts.

Cox regression analysis was used to build the clinical model for PFS prediction. Four clinical features including age, hemoglobin, WBC, and LVSI were found to be correlated with PFS. Inflammation is an important part of the tumor microenvironment and plays a key role in the initiation, promotion, progression, invasion, and metastasis of the tumor (26). Systemic inflammation biomarkers such as C-reactive protein (CRP), platelet count, hemoglobin, and WBC had already been shown to have prognostic values in different tumors (27, 28). In our study, hemoglobin and WBC were also identified as important prognostic factors for PFS which was coherent with the results of the previous studies. In addition, lymph-vascular space invasion is considered to be a crucial factor in the tumor cell dissemination (29) and is identified as an unfavorable prognostic factor in cervical cancer (30, 31), which is consistent with our study.

Radiomics analysis has developed as a non-invasive method to visualize and quantify intra-tumor heterogeneity by high-throughput quantitative characteristic extraction from medical images, thereby providing prognostic information in medical decision making (8). In the recent years, researchers have used radiomics in the prediction of pathological features, LNM, and response to neoadjuvant chemotherapy in cervical cancer (12–14, 32), rather than clinical outcomes such as PFS and overall survival (OS). Jin et al. reported that MRI-based radiomics signature was an independent predictor of DFS in patients with early-stage cervical cancer treated with radical hysterectomy, and their study demonstrated that the Rad-score yielded a C-index of 0.753 (95% CI: 0.696-0.805) on 3-year DFS prediction, which were higher than either clinical model or combined model (33). However, these studies focused on early-stage (IB-IIA) cervical cancer treated by surgery. Only two clinicopathological features (LNM and LVSI) were included in their clinical model, which did not contain another hematological parameter. Some previous studies applied PET/CT radiomics to evaluate patients’ responses to chemoradiotherapy with LACC (34, 35). Although PET/CT demonstrated good discrimination ability, its high cost and high radiation limited its wide use. The role of MRI-based radiomic signatures to evaluate PFS in patients with LACC who have surgical indicators has not yet been investigated. Therefore, in this context, it is necessary for us to do this research.

The LASSO-Cox-based method was used to construct the Rad-score, which derived from the joint T2WI and DWI. Wang et al. have published two articles about using Rad-score based on the joint T2WI and DWI for prediction of LNM or parametrial invasion (PMI) in patients with early cervical cancer (36, 37). These studies demonstrated that the Rad-score from the combined T2WI and DWI has a significant improvement performance for prediction of LNM or PMI, compared with the Rad-score from T2WI or DWI alone. The advantage of T2WI is that it can clearly show the anatomical features of tumors in the cervical cancer patients, and as a functional imaging, DWI can provide microscopic motion of water molecules in tissues and subsequently detect early pathological changes based on water diffusion properties (38). Therefore, the combination of T2WI and DWI could balance the shortcomings and gain more precise and comprehensive information about the tumors. In our study, the radiomic score that combined T2WI and DWI showed good performance for prediction of 3-year PFS in patients with LACC, which yielded a C-index of 0.803 (95% CI, 0.690–0.915) and an AUC of 0.795 (95% CI: 0.653-0.937) in the validation cohort.

To provide a clinically suitable and quantitative approach for the individual prediction of PFS in patients with LACC, a nomogram could enable gynecologists to evaluate the survival of patients based on their clinical and image characteristics. In the current study, a combined nomogram that combined both the Rad-score and other important clinicopathologic features was established for the 3-year PFS evaluation in patients with LACC for the first time. The combined nomogram was shown to have a significant improvement performance for the 3-year PFS prediction, compared with the Rad-score or clinical model alone (C-index of 0.821 vs. 0.778 and 0.816 in the training cohort; 0.829 vs. 0.816 and 0.803 in the validation cohort). This result was also verified by the conclusions of the ROC analysis. It indicated that the radiomics signature may contain information that is complementary to clinical factors, reflecting changes of human tissues at the molecular and genetic levels (9, 33). Another interesting finding of our research was that the Rad-score could serve as a marker in discriminating low-risk and high-risk patients. Patients with higher Rad-scores have the worse PFS. These results provided a new insight into the future treatment protocols in patients with LACC. For instance, LACC patients at high risk of recurrence and metastasis could be considered for preoperative neoadjuvant chemotherapy, whereas patients at low risk of recurrence could directly select surgeries, thereby avoiding unnecessary chemotherapy-related toxicities and disease progression due to delays in effective treatment. Therefore, the Rad-score may be used as an effective biomarker to improve the prognostic ability of pretreatment.

Our research has the following limitations. Firstly, its retrospective design and single-institution study may lead to inevitable selection bias. Prospective multicenter studies with larger populations will be required to confirm the robustness and reproducibility of the current research. Secondly, imaging data were collected by different MR scanners or imaging protocols. Although all the imaging data were normalized to reduce bias before extraction, the performance of the radiomics signature will be significantly improved by normalization of the imaging data. Finally, due to the limited number of patients with genetic data, we did not provide genomic characteristics in our study. In the future study, how to integrate genomic features, radiomics signature, and clinical characteristics together will become increasingly important.

## Conclusion

We developed and validated a Rad-score as a non-invasive method for a preoperative evaluation of PFS in patients with LACC. The combined nomogram, which integrated the Rad-score and clinicopathologic factors, showed significant improvement in the prediction of PFS and may serve as a potential tool to guide individual treatment plans for patients with LACC.

## Data Availability Statement

The raw data supporting the conclusions of this article will be made available by the authors, without undue reservation.

## Ethics Statement

The studies involving human participants were reviewed and approved by the First Affiliated Hospital of Wenzhou Medical University, Wenzhou, China. Written informed consent for participation was not required for this study in accordance with the national legislation and the institutional requirements.

## Author Contributions

MTC wrote the manuscript. MTC, YJY, ZYH, and FL: conception and design. MTC, FY, JD, RRZ, and XWH: collection and assembly of data. ZYH, MTC, FY, JD, RRZ, and XWH: development of methodology. MTC, ZYH, FY: data analysis and interpretation.All authors contributed to the article and approved the submitted version.

## Funding

This paper was financially supported by the Science and Technology Planning Projects of Wenzhou (Grant No. Y20180112), Health Foundation for Creative Talents in Zhejiang Province, China (No: 2016) and Project Foundation for the College Young and Middle-aged Academic Leader of Zhejiang Province, China (No: 2017).

## Conflict of Interest

The authors declare that the research was conducted in the absence of any commercial or financial relationships that could be construed as a potential conflict of interest.

## Publisher’s Note

All claims expressed in this article are solely those of the authors and do not necessarily represent those of their affiliated organizations, or those of the publisher, the editors and the reviewers. Any product that may be evaluated in this article, or claim that may be made by its manufacturer, is not guaranteed or endorsed by the publisher.

## References

[1] Siegel RL Miller KD Jemal A. Cancer Statistics, 2015. CA Cancer J Clin (2015) 65(1):5–29. doi: 10.3322/caac.21254 25559415

[2] Serkies K Jassem J. Systemic Therapy for Cervical Carcinoma - Current Status. Chin J Cancer Res (2018) 30(2):209–21. doi: 10.21147/j.issn.1000-9604.2018.02.04 PMC5953957 29861606

[3] Bhatla N Aoki D Sharma DN Sankaranarayanan R. Cancer of the Cervix Uteri. Int J Gynaecol Obstet (2018) 143(Suppl 2):22–36. doi: 10.1002/ijgo.12611 30306584

[4] Halle MK Ojesina AI Engerud H Woie K Tangen IL Holst F. Clinicopathologic and Molecular Markers in Cervical Carcinoma: A Prospective Cohort Study. Am J Obstet Gynecol (2017) 217(4):432.e1–.e17. doi: 10.1016/j.ajog.2017.05.068 28599900

[5] Rose PG Java J Whitney CW Stehman FB Lanciano R Thomas GM. Nomograms Predicting Progression-Free Survival, Overall Survival, and Pelvic Recurrence in Locally Advanced Cervical Cancer Developed From an Analysis of Identifiable Prognostic Factors in Patients From NRG Oncology/Gynecologic Oncology Group Randomized Trials of Chemoradiotherapy. J Clin Oncol (2015) 33(19):2136–42. doi: 10.1200/jco.2014.57.7122 PMC4477785 25732170

[6] Ng SH Chang TC Ko SF Yen PS Wan YL Tang LM. Nasopharyngeal Carcinoma: MRI and CT Assessment. Neuroradiology (1997) 39(10):741–6. doi: 10.1007/s002340050499 9351114

[7] Kusmirek J Robbins J Allen H Barroilhet L Anderson B Sadowski EA. PET/CT and MRI in the Imaging Assessment of Cervical Cancer. Abdom Imaging (2015) 40(7):2486–511. doi: 10.1007/s00261-015-0363-6 25666968

[8] Gillies RJ Kinahan PE Hricak H. Radiomics: Images Are More Than Pictures, They Are Data. Radiology (2016) 278(2):563–77. doi: 10.1148/radiol.2015151169 PMC4734157 26579733

[9] Aerts HJ Velazquez ER Leijenaar RT Parmar C Grossman P Carvalho S. Decoding Tumour Phenotype by Noninvasive Imaging Using a Quantitative Radiomics Approach. Nat Commun (2014) 5:4006. doi: 10.1038/ncomms5006 24892406 PMC4059926

[10] Lambin P Rios-Velazquez E Leijenaar R Carvalho S van Stiphout RG Granton P. Radiomics: Extracting More Information From Medical Images Using Advanced Feature Analysis. Eur J Cancer (Oxf Engl 1990) (2012) 48(4):441–6. doi: 10.1016/j.ejca.2011.11.036 PMC4533986 22257792

[11] Bartoschek M Oskolkov N Bocci M Lövrot J Larsson C Sommarin M. Spatially and Functionally Distinct Subclasses of Breast Cancer-Associated Fibroblasts Revealed by Single Cell RNA Sequencing. Nat Commun (2018) 9(1):5150. doi: 10.1038/s41467-018-07582-3 30514914 PMC6279758

[12] Erbay G Onal C Karadeli E Guler OC Arica S Koc Z. Predicting Tumor Recurrence in Patients With Cervical Carcinoma Treated With Definitive Chemoradiotherapy: Value of Quantitative Histogram Analysis on Diffusion-Weighted MR Images. Acta Radiol (Stockholm Sweden 1987) (2017) 58(4):481–8. doi: 10.1177/0284185116656492 27445314

[13] Schob S Meyer HJ Pazaitis N Schramm D Bremicker K Exner M. ADC Histogram Analysis of Cervical Cancer Aids Detecting Lymphatic Metastases-A Preliminary Study. Mol Imaging Biol (2017) 19(6):953–62. doi: 10.1007/s11307-017-1073-y 28315203

[14] Liu Y Zhang Y Cheng R Liu S Qu F Yin X. Radiomics Analysis of Apparent Diffusion Coefficient in Cervical Cancer: A Preliminary Study on Histological Grade Evaluation. J Magn Reson Imaging (2019) 49(1):280–90. doi: 10.1002/jmri.26192 29761595

[15] Yushkevich PA Piven J Hazlett HC Smith RG Ho S Gee JC. User-Guided 3D Active Contour Segmentation of Anatomical Structures: Significantly Improved Efficiency and Reliability. NeuroImage (2006) 31(3):1116–28. doi: 10.1016/j.neuroimage.2006.01.015 16545965

[16] Zhang JX Song W Chen ZH Wei JH Liao YJ Lei J. Prognostic and Predictive Value of a microRNA Signature in Stage II Colon Cancer: A microRNA Expression Analysis. Lancet Oncol (2013) 14(13):1295–306. doi: 10.1016/s1470-2045(13)70491-1 24239208

[17] Jiang Y Yuan Q Lv W Xi S Huang W Sun Z. Radiomic Signature of (18)F Fluorodeoxyglucose PET/CT for Prediction of Gastric Cancer Survival and Chemotherapeutic Benefits. Theranostics (2018) 8(21):5915–28. doi: 10.7150/thno.28018 PMC6299427 30613271

[18] Friedman J Hastie T Tibshirani R. Regularization Paths for Generalized Linear Models *via* Coordinate Descent. J Stat Softw (2010) 33(1):1–22. doi: 10.18637/jss.v033.i01 20808728 PMC2929880

[19] Simon N Friedman J Hastie T Tibshirani R. Regularization Paths for Cox’s Proportional Hazards Model *via* Coordinate Descent. J Stat Softw (2011) 39(5):1–13. doi: 10.18637/jss.v039.i05 PMC4824408 27065756

[20] Camp RL Dolled-Filhart M Rimm DL. X-Tile: A New Bio-Informatics Tool for Biomarker Assessment and Outcome-Based Cut-Point Optimization. Clin Cancer Res (2004) 10(21):7252–9. doi: 10.1158/1078-0432.Ccr-04-0713 15534099

[21] Harrell FE Jr. Califf RM Pryor DB Lee KL Rosati RA. Evaluating the Yield of Medical Tests. Jama (1982) 247(18):2543–6. doi: 10.1001/jama.247.18.2543 7069920

[22] Blanche P Kattan MW Gerds TA. The C-Index Is Not Proper for the Evaluation of $T$-Year Predicted Risks. Biostatistics (Oxf Engl) (2019) 20(2):347–57. doi: 10.1093/biostatistics/kxy006 29462286

[23] Heagerty PJ Zheng Y. Survival Model Predictive Accuracy and ROC Curves. Biometrics (2005) 61(1):92–105. doi: 10.1111/j.0006-341X.2005.030814.x 15737082

[24] Chambless LE Diao G. Estimation of Time-Dependent Area Under the ROC Curve for Long-Term Risk Prediction. Stat Med (2006) 25(20):3474–86. doi: 10.1002/sim.2299 16220486

[25] DeLong ER DeLong DM Clarke-Pearson DL. Comparing the Areas Under Two or More Correlated Receiver Operating Characteristic Curves: A Nonparametric Approach. Biometrics (1988) 44(3):837–45. doi: 10.2307/2531595 3203132

[26] Mantovani A Allavena P Sica A Balkwill F. Cancer-Related Inflammation. Nature (2008) 454(7203):436–44. doi: 10.1038/nature07205 18650914

[27] Barkati M Fortin I Mileshkin L Bernshaw D Carrier JF Narayan K. Hemoglobin Level in Cervical Cancer: A Surrogate for an Infiltrative Phenotype. Int J Gynecol Cancer (2013) 23(4):724–9. doi: 10.1097/IGC.0b013e31828a0623 23446376

[28] Wang L Jia J Lin L Guo J Ye X Zheng X. Predictive Value of Hematological Markers of Systemic Inflammation for Managing Cervical Cancer. Oncotarget (2017) 8(27):44824–32. doi: 10.18632/oncotarget.14827 PMC5546522 28148894

[29] Kikuchi E Margulis V Karakiewicz PI Roscigno M Mikami S Lotan Y. Lymphovascular Invasion Predicts Clinical Outcomes in Patients With Node-Negative Upper Tract Urothelial Carcinoma. J Clin Oncol (2009) 27(4):612–8. doi: 10.1200/jco.2008.17.2361 PMC2737380 19075275

[30] Yu Q Lou XM He Y. Prediction of Local Recurrence in Cervical Cancer by a Cox Model Comprised of Lymph Node Status, Lymph-Vascular Space Invasion, and Intratumoral Th17 Cell-Infiltration. Med Oncol (Northwood Lond Engl) (2014) 31(1):795. doi: 10.1007/s12032-013-0795-1 24310812

[31] Twu NF Ou YC Liao CI Chang WY Yang LY Tang YH. Prognostic Factors and Adjuvant Therapy on Survival in Early-Stage Cervical Adenocarcinoma/Adenosquamous Carcinoma After Primary Radical Surgery: A Taiwanese Gynecologic Oncology Group (TGOG) Study. Surg Oncol (2016) 25(3):229–35. doi: 10.1016/j.suronc.2016.05.028 27566027

[32] Downey K Riches SF Morgan VA Giles SL Attygalle AD Ind TE. Relationship Between Imaging Biomarkers of Stage I Cervical Cancer and Poor-Prognosis Histologic Features: Quantitative Histogram Analysis of Diffusion-Weighted MR Images. AJR Am J Roentgenol (2013) 200(2):314–20. doi: 10.2214/ajr.12.9545 23345352

[33] Fang J Zhang B Wang S Jin Y Wang F Ding Y. Association of MRI-Derived Radiomic Biomarker With Disease-Free Survival in Patients With Early-Stage Cervical Cancer. Theranostics (2020) 10(5):2284–92. doi: 10.7150/thno.37429 PMC7019161 32089742

[34] Ho KC Fang YH Chung HW Yen TC Ho TY Chou HH. A Preliminary Investigation Into Textural Features of Intratumoral Metabolic Heterogeneity in (18)F-FDG PET for Overall Survival Prognosis in Patients With Bulky Cervical Cancer Treated With Definitive Concurrent Chemoradiotherapy. Am J Nucl Med Mol Imaging (2016) 6(3):166–75. PMC4965521 27508103

[35] Lucia F Visvikis D Desseroit MC Miranda O Malhaire JP Robin P. Prediction of Outcome Using Pretreatment (18)F-FDG PET/CT and MRI Radiomics in Locally Advanced Cervical Cancer Treated With Chemoradiotherapy. Eur J Nucl Med Mol Imaging (2018) 45(5):768–86. doi: 10.1007/s00259-017-3898-7 29222685

[36] Wang T Gao T Yang J Yan X Wang Y Zhou X. Preoperative Prediction of Pelvic Lymph Nodes Metastasis in Early-Stage Cervical Cancer Using Radiomics Nomogram Developed Based on T2-Weighted MRI and Diffusion-Weighted Imaging. Eur J Radiol (2019) 114:128–35. doi: 10.1016/j.ejrad.2019.01.003 31005162

[37] Wang T Gao T Guo H Wang Y Zhou X Tian J. Preoperative Prediction of Parametrial Invasion in Early-Stage Cervical Cancer With MRI-Based Radiomics Nomogram. Eur Radiol (2020) 30(6):3585–93. doi: 10.1007/s00330-019-06655-1 32065284

[38] Harry VN. Novel Imaging Techniques as Response Biomarkers in Cervical Cancer. Gynecol Oncol (2010) 116(2):253–61. doi: 10.1016/j.ygyno.2009.11.003 20109726

