# Supplementary material for: MRI Radiomic Features: A Potential Biomarker for Progression-Free Survival Prediction of Patients With Locally Advanced Cervical Cancer Undergoing Surgery
Source: Front Oncol. 2021 Dec 14;11:749114. doi: 10.3389/fonc.2021.749114 (PMC8712932; doi:10.3389/fonc.2021.749114)
Supplement: Supplementary file 1 [file DataSheet_1.docx]

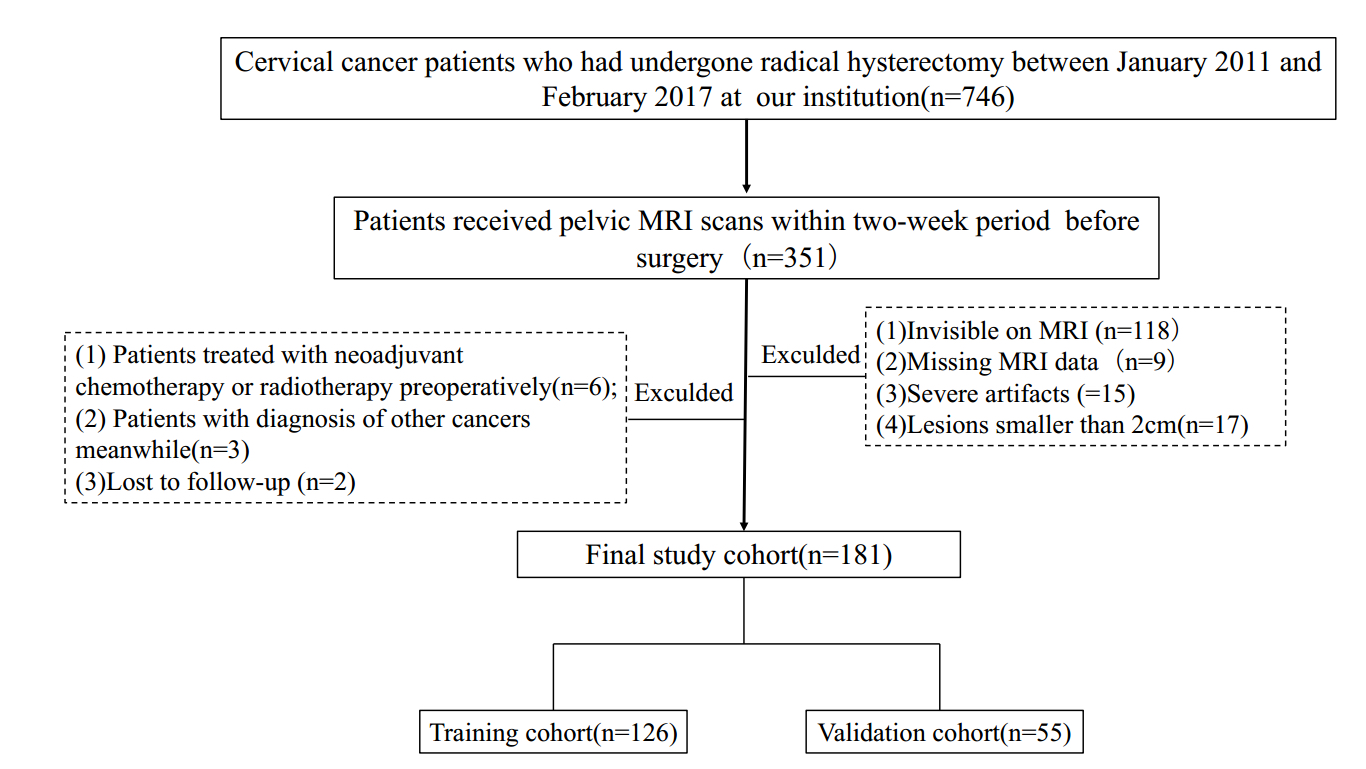


**Figure S1:**The patients’ recruitment workflow.

**
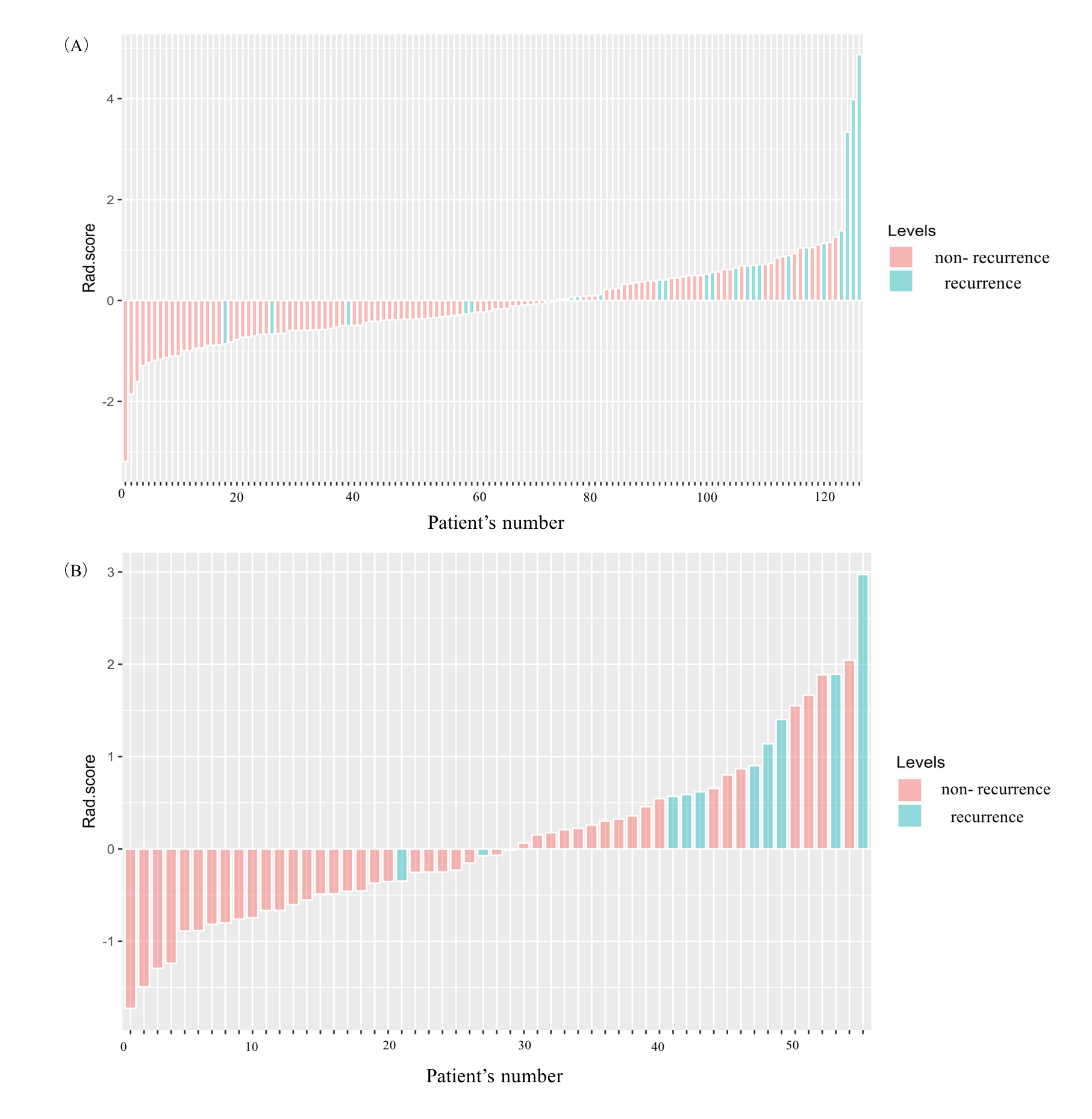
Figure S2:** Distributions of the Rad-score and survival status in the training and validation cohorts, the green marks and red marks indicate the patients in the recurrence (local or distant relapse, metastasis) group and non- recurrence group, respectively, and (A) Rad-score for every patient in the training cohort (N=126). (B) Rad-score for every patient in the validation cohort (N=55).
